# Blocking Palmitoylation of Apelin Receptor Alleviates Morphine Tolerance in Neuropathic Cancer Pain

**DOI:** 10.7150/ijbs.86888

**Published:** 2024-01-01

**Authors:** Xiaoqing Fan, Meiting Gong, Siyu Zhang, Wanxiang Niu, Suling Sun, Huihan Yu, Xueran Chen, Zhiyou Fang

**Affiliations:** 1 Anhui Province Key Laboratory of Medical Physics and Technology; Institute of Health and Medical Technology, Hefei Institutes of Physical Science, Chinese Academy of Sciences, No. 350, Shushan Hu Road, Hefei, Anhui, 230031, China.; 2 Science Island Branch, Graduate School of University of Science and Technology of China, No. 96, Jin Zhai Road, Hefei, Anhui, 230026, China.; 3 Department of Laboratory Medicine, Hefei Cancer Hospital, Chinese Academy of Sciences, No. 350, Shushan Hu Road, Hefei, Anhui, 230031, China.; 4 Department of Anesthesiology, The First Affiliated Hospital of USTC, Division of Life Sciences and Medicine, University of Science and Technology of China (USTC), No. 17, Lu Jiang Road, Hefei, Anhui, 230001, China.; 5 Department of Pathophysiology, School of Basic Medicine, Anhui Medical University, No. 81, Meishan Road, Hefei, Anhui, 230032, China.

**Keywords:** APLNR, ERK1/2, microglial, morphine tolerance, neuropathic cancer pain

## Abstract

Neuropathic cancer pain (NCP) is an important symptom in patients with cancer. However, significant analgesic tolerance and other side effects critically hamper the administration of morphine. Protein palmitoylation mediated by the DHHC family may be involved in the glial activation and inflammatory responses underlying organ failure. In this study, we investigated the key role of protein palmitoylation in cancer pain and sought to target palmitoylation to suppress morphine tolerance. We found that long-term use of morphine led to the accumulation of the morphine metabolite, morphine-3-glucuronide, *in vivo* and activated ERK1/2 and microglia to release inflammatory factors through the apelin receptor APLNR. Palmitoyltransferase ZDHHC9 was upregulated in NCP, and APLNR was palmitylated to protect it from lysosomal degradation and to maintain its stability. We also designed competitive inhibitors of APLNR palmitoylation to inhibit the development of NCP, release of inflammatory factors, and attenuation of morphine tolerance. Therefore, targeting APLNR palmitoylation in combination with morphine is a potent method for cancer pain treatment. Our data provide a basis for the future clinical use of related drugs combined with morphine for the treatment of cancer-related pain.

## Introduction

Pain is one of the most common symptoms of cancer. Cancer-related pain occurs in approximately 33% of patients after curative treatment and in 64% of patients in advanced stages of cancer 1. Approximately one-third of cancer patients suffer from neuropathic cancer pain (NCP); in clinical research, due to the difficulty in controlling cancer pain, NCP accounts for more than half of all pain cases 2,3. NCP often presents as a spontaneous burning sensation, intermittent tingling, or burning warmth, and is more frequent at night. It is a multi-cause, multi-system process that presents with different clinical manifestations at different stages 4. Following nerve injury, input from damaged primary afferent neurons to the dorsal horn of the spinal cord, TLR2 and TLR4 on the surface of glial cells within the dorsal horn of the spinal cord, are significantly activated in a neuropathic pain state. They undergo intracellular signaling through the IRAK1/TRAF6 pathway, activating the transcription factor NF-kB and leading to a significant up-regulation in the expression of the pro-inflammatory cytokines IL-1β, TNF-α, and IL-6. These pro-inflammatory cytokines can further regulate the transcription of inflammatory mediators (including cytokines) through the activation of NF-kB 5. Neuroinflammation is induced by the inflammatory cascade described above. Neuroinflammation, mediated by pro-inflammatory cytokines and chemokines, plays an important role in the formation and maintenance of neuropathic pain. Studies have shown that the development of neuroinflammation can sensitize the neurons responsible for the production and maintenance of nociception, leading to the onset and persistence of pain 6. Currently, there is a lack of effective strategies for the treatment of neuropathic pain; therefore, a detailed study on the mechanism of NCP is needed to explore different treatment methods for effective clinical pain control and relief, as well as improving patients' quality of life.

Most patients require opioids, which are recommended for controlling moderate-to-severe NCP 7,8. Morphine is one of the most commonly used drugs for the treatment of postoperative and cancer pain. Long durations of μ-receptor desensitization and cellular adaptation mediated by the G protein-coupling signaling pathway may occur after long-term morphine use 9,10. Even if morphine is used in large quantities over a long period of time, activate glial cells can be activated via other receptors in the G protein-coupled receptor (GPCR) family, producing an inflammatory response 11,12. Prolonged morphine use also produces powerful microglial changes, manifested as cell hypertrophy and increased microglial CD11b and Iba1 expression 13,14. Pro-inflammatory cytokines, particularly interleukin (IL-1β), tumor necrosis factor-α (TNF-α), and IL-17, further induce the activation of protein kinase C in neurons, activating the sodium and calcium channels, and adenylate cyclase, leading to central sensitization and weakened analgesic effects 15,16. This may play a key role in the loss of the analgesic effects of opioids. Clinically, ketamine has been used in combination with morphine to treat refractory cancer pain. Low-dose ketamine has anti-hyperalgesic and anti-inflammatory effects, but these effects are only temporary 17,18. Therefore, there is an urgent need to identify novel cancer treatments for cancer pain.

Palmitoylation is a post-translational modification catalyzed by 23 palmitoyltransferase families containing the Asp-His-His-Cys (DHHC) domain, which has recently emerged as a critical modulator of neuronal function 19. Abnormalities in the DHHC proteins and palmitoylation are associated with neurological abnormalities in humans 20,21. H-Ras palmitoylation, mediated by acyl protein thioesterase-1, affects plasma membrane localization, which activates the Ras signaling pathway, thus stimulating microglial proliferation and inflammatory cytokine production 22. Hyperpalmitoylated glial fibrillary acidic protein promotes astrocyte proliferation *in vivo*, culminating in astrocyte proliferation in infant neuronal ceroid lipofuscin 23.

In this study, we used a mouse model of NCP to explore the mechanism of morphine tolerance and found that long-term use of morphine led to *in vivo* accumulation of the morphine metabolite morphine-3-glucuronide (M3G). Furthermore, it activates extracellular signal-regulated protein kinase (ERK)1/2 and microglia through the apelin receptor (APLNR) to release inflammatory factors. Further studies showed that the expression of palmitoyltransferase ZDHHC9 was upregulated in the presence of neuropathic pain. Palmitylated APLNR prevented the degradation of palmitoyltransferase ZDHHC9 by lysosomes and promoted the stability of the protein in cells. Finally, we designed competitive peptides targeting the palmitoylated site of APLNR to treat cancer pain in combination with morphine and achieved good results. These findings reveal the mechanism of morphine tolerance and provide new ideas for the clinical treatment of cancer pain.

## Materials and Methods

### Animals

All procedures in this study were approved by the Ethics Committee of Science and Technology of the Hefei Institute of Physical Science, Chinese Academy of Sciences and conformed to the animal standards involved in the ARRIVE guidelines. C57BL/6 mice (50% male, 50% female) weighing 25-30 g were purchased from Chengdu Yaokang Biotechnology Co., Ltd. (Sichuan, China) and maintained under a 12:12-h light/dark cycle. All animal behavioral tests were conducted from 8 am to 10 am.

### NCP Model

The NCP model was established as described previously 24,25. Well-grown mouse sarcoma S-180 cells were inoculated into the peritoneal cavity of mice. After 7 days of culture, mice inoculated with tumor cells were anesthetized with pentobarbital (50 mg/kg) and ascites were extracted. The tumor source was adjusted to 1×10^7^ cells/ml with a serum-free medium, and 0.2 ml (2×10^6^ cells) was inoculated into the muscle tissue near the nerve close to the rotor immediately after the distal biceps femoris semitendinosus muscle of the rotor branches out from the common sciatic nerve in the right leg. Sham operations were performed on the other mice. For the sham operation, aspirated ascites were centrifuged at 10,000 rpm for 10 min, and the supernatant was collected to separate the liquid component from the tumor cells. Similarly, the same volume of supernatant was injected into the ascites instead of the tumor cells. Magnetic resonance imaging (MRI) was performed to confirm the presence of a tumor around the sciatic nerve by anatomical examination.

### Intrathecal injection procedure

Mice were injected intrathecally with 0.9% physiological saline (10 µl) or morphine (10 µg/ul) twice daily for 6 days with or without ketamine (50 µg/10 µl) or the competitive peptide inhibitor APLNR-S1 [50 µg/10 µl, R(9)-ACTSMLCCGQSRCAG] which was linked to transmembrane peptides.

Intrathecal injections were performed as previously described 26. Briefly, the mouse was placed in the supine position and the midpoint between the tips of the iliac crest was identified. A Hamilton syringe with a 30-gauge needle was inserted into the subarachnoid space of the spinal cord between the L5 and L6 spinous processes. The correct intrathecal injection was confirmed by observing the tail flap. Intrathecal injection did not affect the baseline response compared with the latency recorded before the injection.

### Evaluation of spontaneous persistent pain

Spontaneous pain behavior in mice was assessed as described previously 27, with some modifications. Lifting of diseased paws was the hallmark event of spontaneous pain, which was assessed before and after inoculation in both the sham and NCP mouse models. After 15 min of acclimation in the test chamber, the animals were placed individually in a 19×29×13 cm clear-bottom cage for 30 min and observed by two scorekeepers. The position of each hind paw was divided into the following categories:0, normal weight bearing; 1, light weight bearing; 2, contact with the ground only with the edge of the paw; 3, paw almost lifted from the ground; 4, paw completely lifted; 5, licking of the lifted paw, and the time spent in each position was recorded. For each minute observed, a weighted pain score was calculated (t1 +2t2 +...5t5, where tx is the time spent in category x), and the average of the 6-minute observations was calculated.

### Mechanical threshold measurements with von Frey filaments

The animals were acclimated to the test environment for 2 days. One hour after morphine administration, the mechanical thresholds were measured using von Frey filaments. Briefly, mice were placed in a transparent box and acclimated to the environment for 30 min. The experiments were evaluated by two independent experimenters who had no prior knowledge of the characteristics of the mice. Mechanical pain abnormalities were assessed using the "up-and-down method" 28. Briefly, von Frey filaments with incremental stiffness (bending force of 0.1-2.0 g) were applied to the hind paws for 5 seconds. The 1-g filament was the first to appear; if the animal retracted its paw, it was given a finer filament. If the animal did not retract its paw, a stiffer filament appeared; the cut-off value for the longer filament was 2 g. Measurements were taken before S-180 inoculation (baseline), on specified days after inoculation, and after drug treatment. The claw reduction thresholds (PWT) were calculated after completing a series of six consecutive responses. Data are expressed in grams and represent mean thresholds. The anti-injury response was calculated as a percentage of the maximum possible effect (%MPE) using the following formula:100% × ([PWT after administration-PWT before administration]/[2 g PWT before administration] ) = %MPE.

### Palmitoylation determination

Protein palmitoyl modifications by acylbiotinyl exchange (ABE) were analyzed as previously described 29. Cell lysates were prepared in the presence of 10 mM N-ethylmaleimide (23030; Pierce, CA, USA) and denatured in chloroform/methanol. Samples were incubated overnight with 10 mM N-ethylmaleimide. After washing with chloroform/methanol, the samples were treated with 1M hydroxylamine (HAM). After each chloroform/methanol precipitation step, the protein pellet was dissolved in an aqueous buffer by sonication. After washing, 5 µM HPDP-biotin (A35390; Pierce) was used for sulfhydryl biotinylation. Affinity purification of the biotin-exchanged samples was performed using the NeutrAvidin resin (53151; Pierce). Purified proteins were eluted by boiling in non-reducing sodium dodecyl sulfate-polyacrylamide gel electrophoresis loading buffer and either analyzed using liquid chromatography-tandem mass spectrometry (LC-MS/MS) or detected using neutral anti-biotin proteins coupled to horseradish peroxidase.

### Statistical Analysis

All grouped data are expressed as mean ± standard error. GraphPad Prism 6 software (GraphPad Software, San Diego, CA, USA) was used for statistical analyses. Differences between the two groups were assessed using Student's *t* test. Data from more than two groups were evaluated using one-way analysis of variance (ANOVA) followed by Tukey's multiple comparison test or two-way ANOVA followed by Bonferroni's post-hoc test. All experiments were repeated for each specimen with at least five biological replicates. The group size (n), where n is the number of independent values in different experiments for each group *in vivo* and *in vitro*, is reported in the figure legends. Significance criteria (p-values) were set as shown in the figure. Statistical significance was defined as *p < 0.05, **p < 0.01, and ***p < 0.001.

## Results

### Morphine could not inhibit cancer pain for a long time

**Figure 1:**
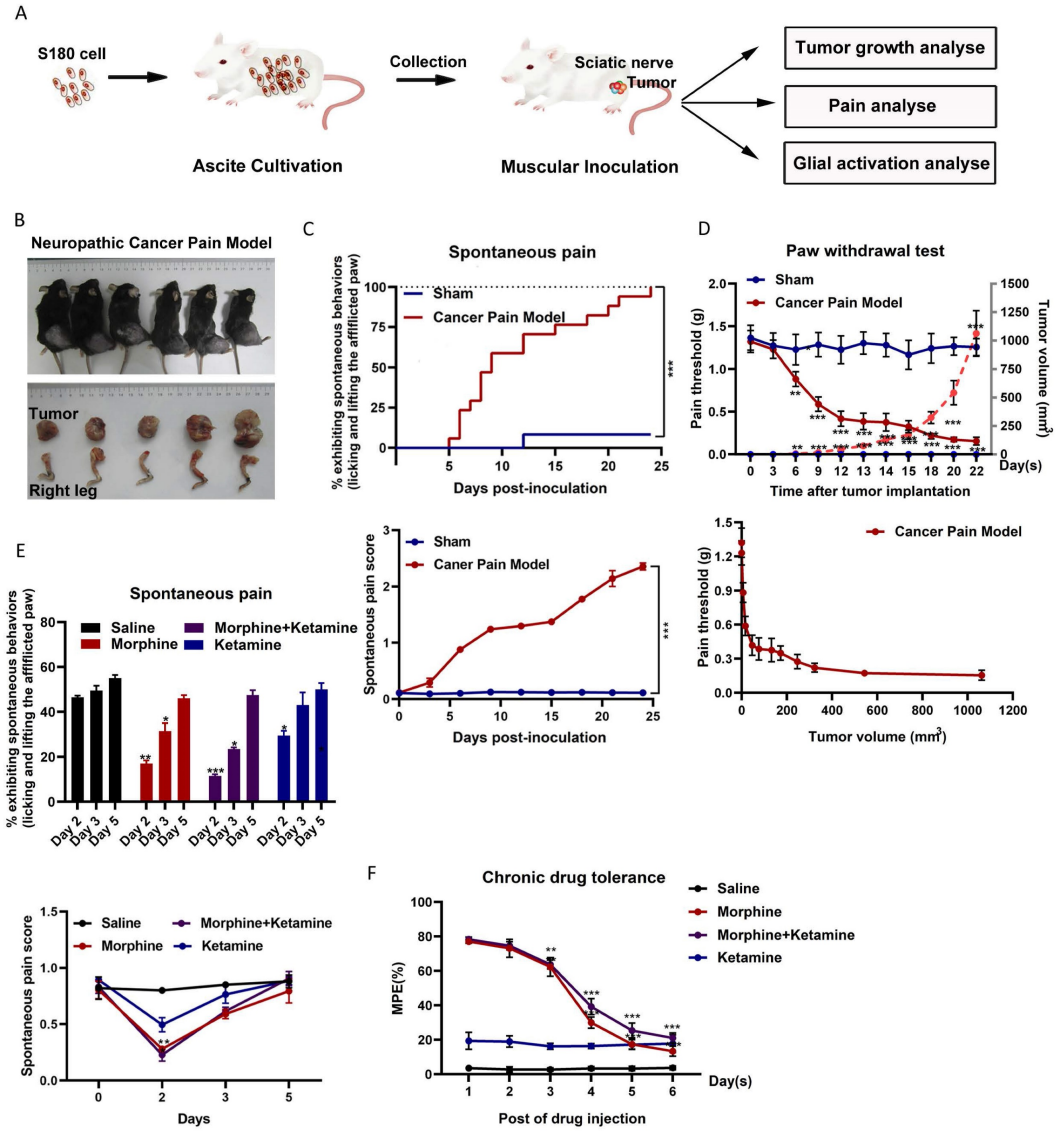
** Spontaneous pain occurred, and ketamine did not suppress chronic morphine tolerance in a neuropathic cancer pain (NCP) mouse model.** (A) Experimental scheme for the NCP model. (B) Dissection of the right leg with the invasive tumor tissue (bottom) and the resulting NCP (top) on day 22 post-inoculation. (C) Time course of spontaneous pain after inoculation in the sham and NCP models (top). The spontaneous pain score was calculated from observations of paw favoring, lifting, and licking before and after inoculation (bottom panel). N=18 mice per group (50% male, 50% female). (D) The withdrawal latency of the right hind paw and tumor volume were measured at the indicated times after inoculation (top). An analysis of the relationship between the paw withdrawal threshold (PWT) and tumor growth rate is presented (bottom). N=10 mice per group (50% male, 50% female). (E) On days 2, 3, and 5 after injection, spontaneous pain was examined to determine the chronic analgesic effect (top). The spontaneous pain score was calculated from observations of paws favoring, lifting, and licking before and on day 2, 3, and 5 after drug injection (bottom). N=5 mice per group (2 males, 3 females). (F) The co-administration of ketamine and morphine did not suppress chronic morphine tolerance. Morphine (10 µg) was intrathecally injected with ketamine (50 µg) twice daily, and the maximal possible effect was measured 1 h after the first injection each day. N=5 mice per group (two males and three females).

The relationship between tumor growth and NCP occurrence was analyzed (Figure 1A). After inoculation, the number of S-180 tumor cells rapidly increased in a time-dependent manner around the sciatic nerve (Figures 1B). Intraplantar inoculation of S-180 cells, but not the vehicle (supernatant of aspirated ascites), in C57BL/6 mice induced pain on post-inoculation day 3, which became more pronounced after day 6 (Figure 1C). On post-inoculation day 6, signs of sciatic nerve damage were observed. As the tumor grew, the mice gradually demonstrated sensory impairment and frequently showed spontaneous pain symptoms, such as lifting of the afflicted right hind limb.

Consistent with this finding, intraplantar inoculation of S-180 cells induced mechanical hyperalgesia (Figure 1D). As the tumor grew, the PWT dropped from 1.32 ± 0.113 g before sarcoma inoculation to 0.154 ± 0.106 g at 1062.85 ± 232.75 mm^3^ (post-inoculation day 22). The PWT for tumor volume from 50 mm^3^ (around day 12) to 250 mm^3^ (around day 18) was maintained at 0.4 g. Thus, the time window for tumor growth should be selected for follow-up studies.

Next, we evaluated the effect of morphine on NCP. On the fifth day of morphine treatment (twice per day for 2-6 days), morphine did not effectively inhibit the occurrence of pain (Figure 1E) and produced a tolerance response (Figure 1F). Ketamine only slightly altered the pain threshold after administration. Co-administration of ketamine did not alter chronic morphine tolerance.

### M3G activated microglia through APLNR

**Figure 2:**
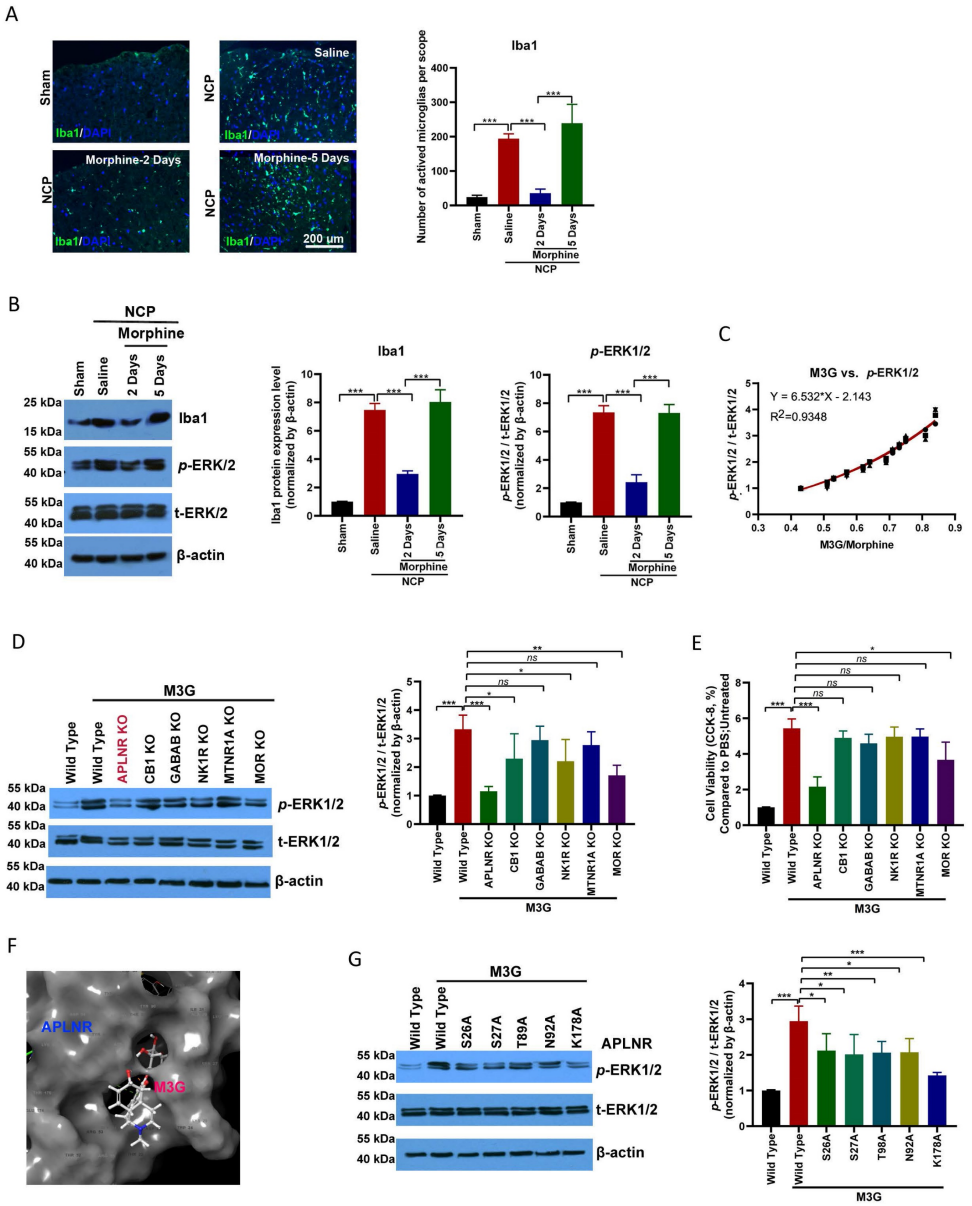
** Morphine-3-glucuronide (M3G) interacts with APLNR.** (A) Immunofluorescence analysis of IBA1 expression in the L4-6 spinal cord horn of the sham and neuropathic cancer pain (NCP) groups. Morphine (10 µg) was injected into the mice twice daily, and spinal samples were collected 2 and 5 days after the final injection to explore chronic morphine tolerance. Scale bar, 200 μm. The number of activated microglia (Iba1-positive cells) in a 20x field of view was quantified. Data represent means ± SEM of three independent experiments. (B) Western blot analysis of IBA1 and *p*-extracellular signal‑regulated protein kinase (ERK)1/2 in the L4-6 spinal cord horn of the sham and NCP groups. The expression of* p*-ERK1/2 was quantified. Data represent means ± SEM of three independent experiments. (C) ERK1/2 phosphorylation is positively associated with morphine-3-glucuronide (M3G)/morphine levels in the mouse NCP model. In the chronic morphine tolerance experiment, the M3G/morphine ratio was analyzed using liquid chromatography-triple quadrupole mass spectrometry (LC-MS/MS). N=12 mice per group (50% male, 50% female). (D) Western blot analysis of *p*-ERK1/2 in M3G (5 μM)-treated microglia BV2 cells with wild type, APLNR knockout, CB1 knockout, GABAB knockout, NK1R knockout, MTNR1A knockout, or MOR knockout. The expression level of *p*-ERK1/2 was quantified. Data represent means ± SEM of three independent experiments. (E) Cell Counting Kit-8 (CCK-8) analysis of cell viability of M3G (5 μM)-treated microglia BV2 cells with wild-type, APLNR knockout, CB1 knockout, GABAB knockout, NK1R knockout, MTNR1A knockout, or MOR knockout. N=3/each group. (F) Cartoon-and-stick representation of the APLNR and M3G binding mode molecular docking was performed using the ZDOCK server. (G) Western blot analysis of *p*-ERK1/2 in M3G (5 μM)-treated microglial BV2 cells with wild-type, APLNR S26A, APLNR S27A, APLNR T98A, APLNR N92A, or APLNR K178A. The expression of *p*-ERK1/2 was quantified. Data represent means ± SEM of three independent experiments.

Microglial activation was significantly enhanced in the cancer pain model group compared to the control group. Morphine administration inhibited microglial activation; however, long-term continuous morphine administration led to microglial reactivation (Figure 2A). IBA1 expression and ERK1/2, a microglia activation-related signaling pathway, activation were inhibited on the second day of morphine administration but re-activated on the fifth day of morphine treatment (Figure 2B).

In the chronic analgesic effect assay, M3G, the main metabolite of morphine, accumulated in the cancer pain model and activated ERK1/2 signal transduction (Figure 2C). Interestingly, we found that M3G activated ERK1/2 through APLNR, in addition to morphine and mu-opioid receptors (MOR). GPCR family receptors were knocked out separately and we found that M3G did not activate ERK1/2 after APLNR or MOR knockout (Figure 2D). Consistent with this, M3G was found to regulate microglial viability through APLNR (Figure 2E).

Molecular docking was used to resolve the key sites of M3G-APLNR binding interactions using the ZDOCK server. The interaction between APLNR and M3G is shown in Figure 2F. The residues, Ser-26, Ser-27, Thr-89, Asn-92, and Lys-178, in APLNR bind with M3G through hydrogen bonding, hydrophobic, and cation-π interactions, respectively. Therefore, we designed and constructed a batch of APLNR mutant plasmids to analyze the effect of these loci on the affinity of M3G for APLNR and the activation of ERK1/2 (Figure 2G). These results suggest that M3G-APLNR interaction may be mostly attributed to the APLNR residues Ser-26, Ser-27, Thr-89, Asn-92, and Lys-178, which contribute to ERK1/2 activation.

Long-term and large-scale use of morphine may cause M3G *in vivo* accumulation and exacerbate ERK1/2 activation through APLNR.

### APLNR palmitoylation occurred in the NCP model

**Figure 3:**
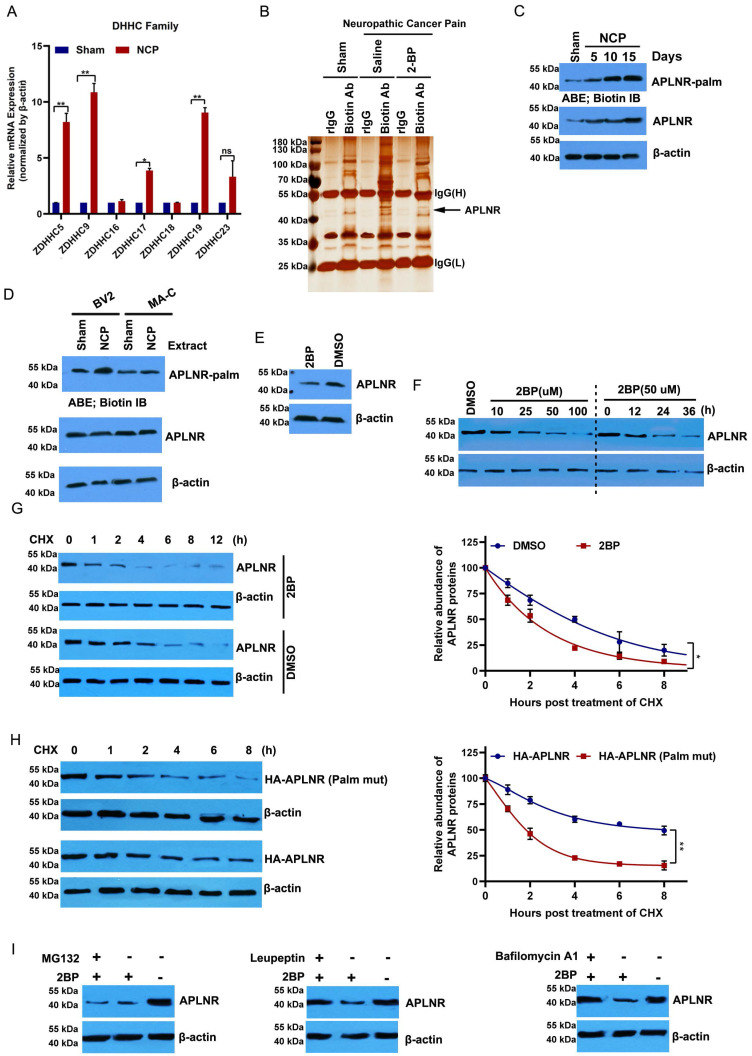
** APLNR palmitoylation occurs in a mouse neuropathic cancer pain (NCP) model and is critical to the stability of APLNR protein.** (A) Real-time polymerase chain reaction analysis of DHHC mRNA levels in the L4-L6 spinal cord horn of NCP model (12 days post-inoculation) and sham mice. β-actin was used as the loading control. N=3/ group. (B) LC-MS/MS analysis of palmitoylated proteins in the L4-L6 spinal cord horn of the NCP model (12 days post-inoculation), sham mouse group, and 2-bromopalmitate (2-BP)-administered NCP model. Lysates from the L4-L6 spinal cord horn from the sham group, NCP model, and 2-BP-administered NCP model (6 h after 2-BP administration) were subjected to the acyl-biotinyl exchange (ABE) method with a streptavidin-biotinylated antibody and then subjected to LC-MS/MS. The identified peptide sequences of APLNR are shown. (C) ABE analysis of APLNR palmitoylation in the L4-L6 spinal cord horn of the NCP model (at days 5, 10, and 15 post-inoculation) and sham mice. Palm, palmitoyaltion. (D) ABE analysis of palmitoylation of APLNR in microglia BV2, and MA-C astrocytes treated with extracts from the L4-L6 spinal dorsal horn of the NCP model for 16h. (E) Western blot analysis of APLNR protein expression in BV2 cells treated with 50 μM 2-BP for 24 h. (F) Western blot analysis of APLNR protein expression in BV2 treated with 2-BP at different doses for 24 h (left panel) or 50 μM for different durations (right). (G) Western blot analysis of APLNR protein expression in BV2 cells treated with 50 μM 2-BP or equivalent dimethyl sulfoxide for 24 h and subsequently subjected to cycloheximide (CHX) chase assay (left panel). APLNR protein levels normalized to β-actin are presented relative to the level (set as 100) 0 h after CHX treatment (right panel). APLNR expression was quantified. Data represented as means ± SEM of three independent experiments. (H) Western blot analysis of APLNR protein expression in BV2 cells transfected with plasmids expressing HA-APLNR or HA-APLNR C325 and 326A for 36 h, and subsequently subjected to a cycloheximide (CHX) chase assay. APLNR expression was quantified. Data represented as means ± SEM of three independent experiments. (I) Western blot analysis of APLNR protein expression in BV2 cells treated with 50 μM 2-BP in the presence of 50 μM leupeptin, 100 nM bafilomycin A1 for 24 h, or 2-BP for 20 h, followed by treatment with 40 μM MG132 for 4 h.

Protein palmitoylation, which is mediated by the DHHC family, plays an important role in glial activation and inflammatory cytokine release 29,30. Of the 23 protein acyltransferases, seven are linked to neurodevelopment and the inflammatory response 21,31. ZDHHC5, 9, 17, and 19 were significantly upregulated in the cancer pain model compared to the sham group (Figure 3A). Then, we purified palmitoylated proteins from the L4-L6 spinal dorsal horn of the cancer pain model using a streptavidin biotinylation antibody and the acyl biotin exchange method (ABE) and analyzed their components using LC-MS/MS. APLNR was higher in the cancer pain model group than in the sham operation group; however, administration of 2-BP, a palmitate analog, attenuated this increase (Figure 3B). In line with this, the palmitylation level of APLNR in the cancer pain model group was higher than that in the sham operation group and positively correlated with the duration of NCP (Figure 3C). Notably, we found that APLNR palmitoylation occurred specifically in microglial cells treated with extracts from the L4-L6 spinal dorsal horns of the NCP model rather than in astrocytes (Figure 3D). These findings suggest that the DHHC-mediated palmitylation of APLNR plays an important role in NCP development.

### Palmitoylation of APLNR is critical to the stability of APLNR protein

2-BP treatment significantly inhibited APLNR protein expression in a dose- and time-dependent manner (Figure 3E and F). We also monitored the degradation kinetics of endogenous APLNR after 2-BP treatment using cycloheximide (CHX) chase analysis. We found that APLNR protein levels decreased rapidly at the onset of CHX treatment. Its half-life was reduced to 2 h in 2-BP-treated cells and 4 h in dimethyl sulfoxide-treated cells (Figure 3G). To confirm these results, the stability of the wild-type and non-palmitoylated APLNR was analyzed using HA labelling. Compared to HA-APLNR, palmitoylated mutant HA-APLNR proteins were degraded rapidly, with half-lives of 2 and 8 h, respectively (Figure 3H). These data confirm that palmitylation is important for the stability of APLNR proteins.

The APLNR degradation pathway under palmitoyl deficiency was further investigated. The inhibition of the lysosomal pathway by leukopeptide and baffamycin A1 significantly prevented 2-BP-induced APLNR degradation in BV2 microglia (Figure 3I). Inhibition of the ubiquitin-proteasome pathway by the proteasome inhibitor MG132 did not prevent APLNR degradation, suggesting that APLNR was degraded by the lysosomal pathway after 2-BP treatment.

### APLNR palmitoylation mediated by ZDHHC9 promoted microglial cell activation

**Figure 4:**
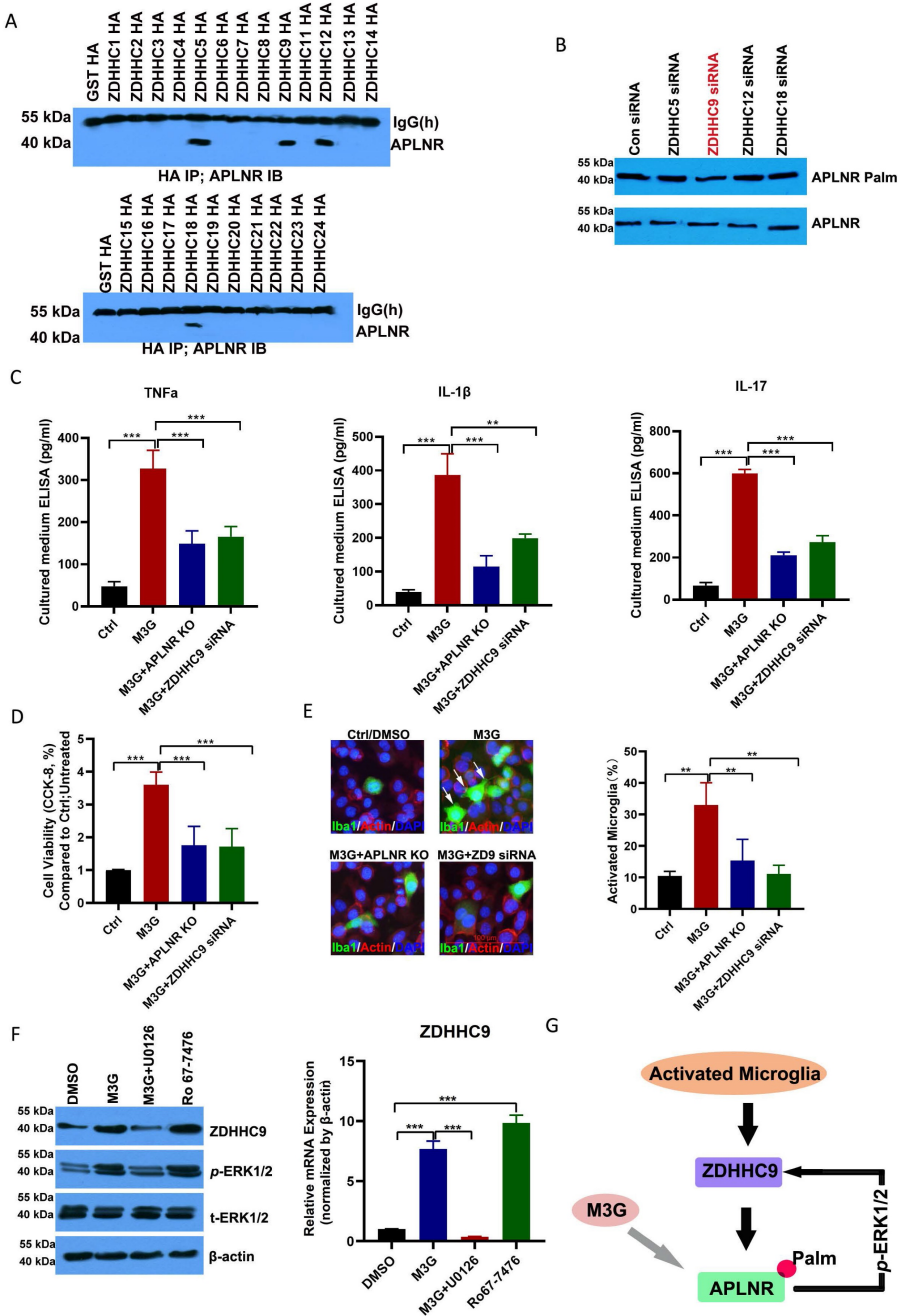
** APLNR palmitoylation mediated by ZDHHC9 activates microglia.** (A) Immunoprecipitation analysis of the interactions between APLNR and DHHCs. BV2 cells were transfected with constructs encoding HA-ZDHHCs in a six-well plate. Cell lysates were harvested for immunoprecipitation using an anti-HA antibody and western blot analysis using an APLNR antibody. (B) ABE analysis of APLNR palmitoylation in BV2 cells infected with ZDHHC5, 9, 12, or 18 siRNAs for 48 h. (C) Enzyme-linked immunosorbent assay (ELISA) analysis of inflammatory cytokines, TNF-α, IL-1β, and IL-17, in morphine-3-glucuronide (M3G) (5 μM)-treated microglia BV2 cells with wild-type, APLNR knockout, or ZDHHC9 knockdown. N=5/group. (D) CCK-8 analysis of cell viability of M3G (5 μM)-treated microglia BV2 cells with wild-type, APLNR knockout, or ZDHHC9 knockdown. N=3/group. (E) Immunofluorescence analysis of IBA1 expression in M3G (5 μM)-treated microglial BV2 cells with wild type, APLNR knockout, or ZDHHC9 knockdown. The ratio of the number of activated microglia (IBA1-positive cells) to the total number of cells (DAPI-positive cells) in a 20x field of view was quantified. Data represented as means ± SEM of three independent experiments. (F) Western blot analysis of ZDHHC9 and *p*-ERK1/2 expression in dimethyl sulfoxide, M3G (5 μM), M3G (5 μM)+U0126 (10 μM), or Ro67-7476 (10 μM) treated BV2 cells. (G) Schematic of M3G activation of ERK1/2 through the ZDHHC9-APLNR axis in a positive feedback regulatory loop.

Immunoprecipitation demonstrated that APLNR was associated with ZDHHC5, ZDHHC9, ZDHHC12, and ZDHHC18 (Figure 4A and Supplementary Figure 1). Notably, ZDHHC9 knockdown substantially decreased APLNR palmitoylation (Figures 4B); therefore, ZDHHC9 is considered a potential enzyme that interacts with palmitoylated APLNR.

M3G promoted the release of inflammatory factors TNF-α, IL-1β, and IL-17 in microglia BV2 cells; however, APLNR knockout or ZDHHC9 knockdown inhibited the release of these inflammatory factors (Figure 4C). Consistent with this, M3G also promoted the viability of microglial cells (Figure 4D) and activated microglia (Figure 4E); however, these effects were suppressed in APLNR-knockout and ZDHHC9-knockdown microglial cells.

M3G increased the expression of ZDHHC9, which may be due to the activation of ERK1/2. The *p*-ERK1/2 inhibitor U0126 inhibited the increased expression of ZDHHC9 caused by M3G, whereas the *p*-ERK1/2 agonist Ro-7476 increased the expression of ZDHHC9 (Figure 4F). This indicates that in the long-term treatment of NCP with morphine there is a positive feedback regulatory mechanism in which M3G activates ERK1/2 through APLNR, and ERK1/2 further improves the expression of ZDHHC9 and APLNR stability (Figure 4G). Finally, as the inflammatory response increases, morphine resistance is enhanced, and the treatment effect on cancer pain is poor.

### Targeting APLNR palmitoylation with a peptidic inhibitor

**Figure 5:**
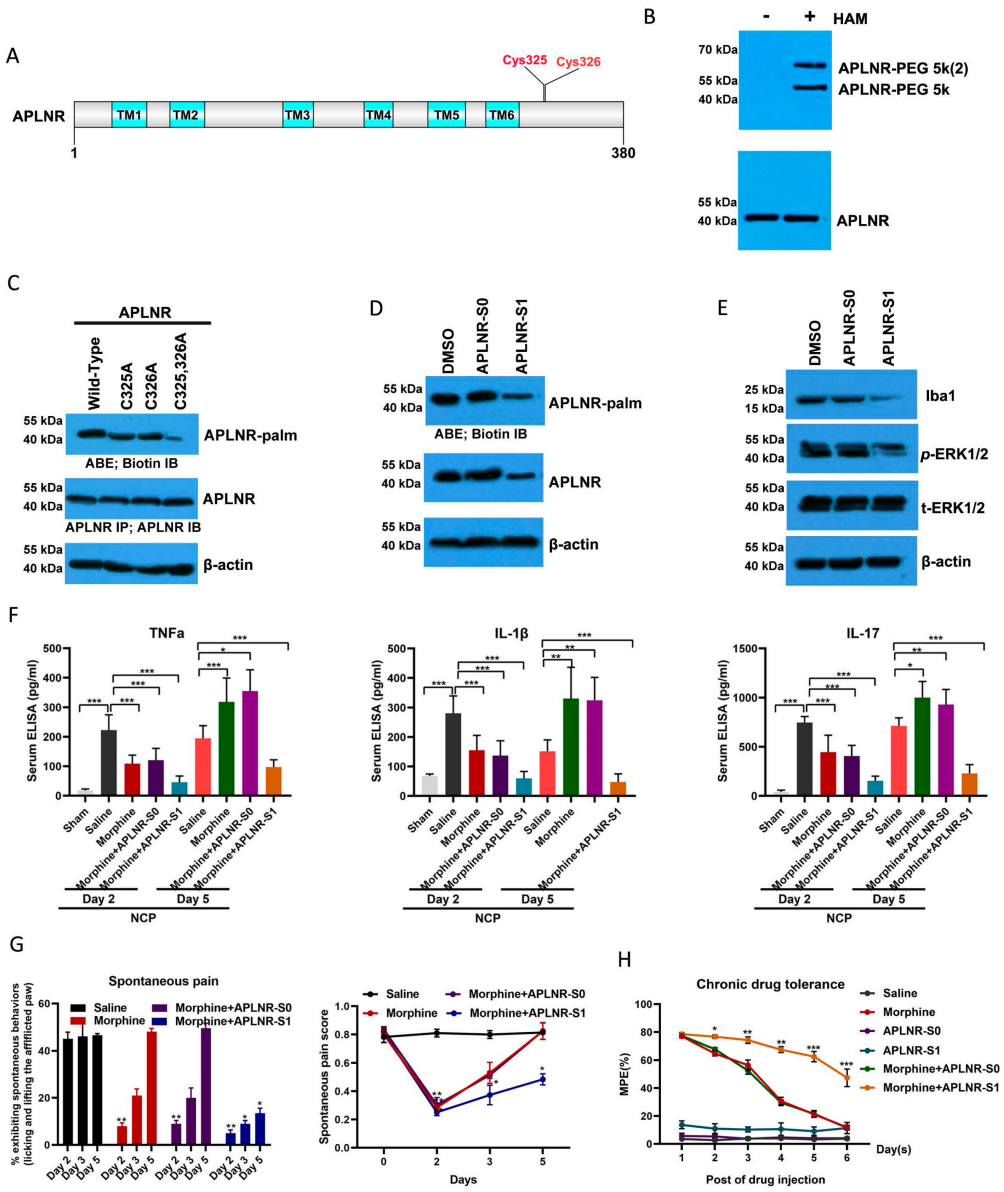
** Targeting APLNR palmitoylation with a peptidic inhibitor.** (A) Schematic representation of potential palmitoylation sites of APLNR protein. (B) ABE analysis of APLNR palmitoylation in microglial BV2 cells using biotin-PEG-5k. HAM, Hydroxylamine. (C) ABE analysis of APLNR palmitoylation in wild-type, APLNR C325A, APLNR C326A, and APLNR C325 and 326A microglial BV2 cells. (D) ABE analysis of APLNR palmitoylation in microglial BV2 cells treated with APLNR-S0 (20 μg/ml) or APLNR-S1 (20 μg/ml). (E) Western blot analysis of IBA1 and *p*-ERK1/2 expression in microglial BV2 cells treated with APLNR-S0 (20 μg/ml) or APLNR-S1 (20 μg/ml). (F) ELISA analysis of the inflammatory cytokines, TNF-α, IL-1β, and IL-17, in the L4-6 spinal cord horn of the NCP and sham groups. Morphine (10 µg) with or without APLNR-S0 (50 μg) or APLNR-S1 (50 μg) was injected into the mice twice daily, and spinal samples were collected 2 and 5 days after the final administration for the chronic morphine tolerance protocol. N=15 mice per group (7 males, 8 females). (G) Time course of spontaneous pain in the sham and NCP groups (left). The spontaneous pain score was calculated from observations of paw favoring, lifting, and licking before and after administration (right). Morphine (10 µg) with or without APLNR-S0 (50 μg) or APLNR-S1 (50 μg) was injected into mice twice daily, and spontaneous pain was examined 2, 3, and 5 days after the final chronic morphine tolerance experiments. N=5 mice/group (2 males, 3 females). (H) The withdrawal latency of the right hind paw and tumor volume were measured at the indicated time points after administration. N=5 mice/group (2 males, 3 females).

Using online software (CSS-Palm: csspalm.biocuckoo.org), we analyzed and identified two potential palmitoylation sites in the APLNR protein (Cys325 and Cys326; Figure 5A). The results of the ABE assay with biotin-PEG (5k) also revealed that APLNR has two palmitoylation sites (Figure 5B), and that the mutation of Cys325 and Cys326 to Ala could abolish APLNR palmitoylation (Figure 5C).

After determining the palmitoylation motif of APLNR, we introduced a competitive inhibitor of APLNR palmitoylation into the NCP model. A cell-penetrating peptide containing the APLNR(319-333) palmitylation sequence (APLNR-S1) and a control peptide containing Cys325 and Cys326 Ala mutations (APLNR-S0) were synthesized *in vitro*. Additionally, the ABE assay showed that APLNR-S1 reduced the palmitoylation and expression of APLNR (Figure 5D). Importantly, the expression of IBA1 and activation of ERK1/2 activation were also weakened (Figure 5E).

Next, we tested the efficacy of the competing peptide, APLNR-S1, in the treatment of cancer pain. On the second and third days of continuous administration, morphine or the combination of morphine and APLNR-S0 significantly inhibited the release of inflammatory factors TNF-α, IL-1β, and IL-17 and the incidence of pain; however, on the fifth day, morphine or the combination of morphine and APLNR-S0 produced drug resistance, and treatment failure occurred (Figure 5F and 5G). However, the combination of morphine and APLNR-S1 resulted in better inhibition of the release of inflammatory factors and pain incidence on the second, third, and fifth days of continuous administration. Consistent with this, MPE results showed that APLNR-S1 significantly attenuated chronic morphine tolerance (Figure 5H).

**Figure 6:**
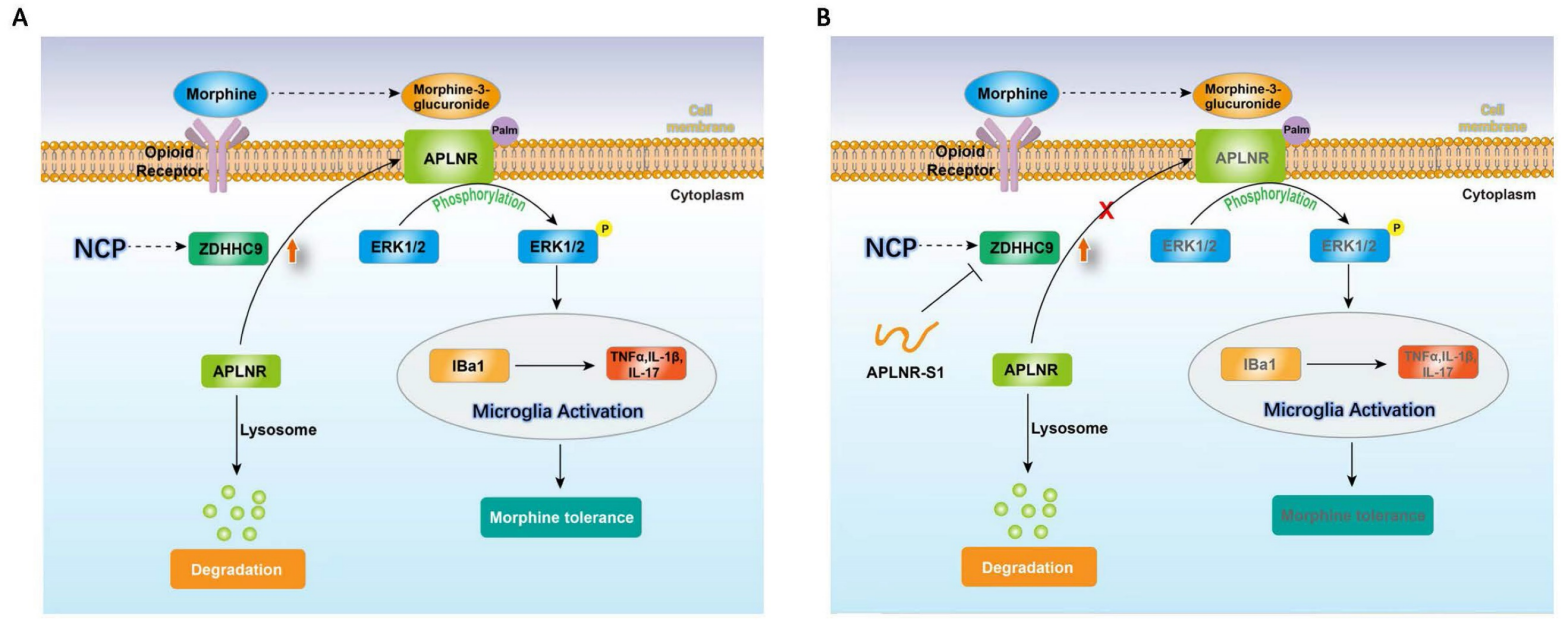
** Illustration of the crosstalk between APLNR palmitoylation and neuropathic cancer pain (NCP).** During the occurrence and development of NCP, the expression of palmityltransferase ZDHHC9 increased, and palmitylated APLNR promoted its stability. The use of morphine can lead to the accumulation of the metabolite M3G *in vivo*, activating the ERK1/2 signaling pathway through APLNR, leading to the activation of microglia, release of inflammatory factors and, finally, formation of morphine tolerance.

Therefore, long-term morphine injection leads to the accumulation of M3G, which in turn activates APLNR and the inflammatory response. APLNR stability is effectively inhibited by targeting palmitoylation of the competitive peptide APLNR-S1. Combined with morphine, it is an effective means of treating neuropathic pain and provides new ideas and schemes for the clinical treatment of cancer pain (Figure 6).

## Discussion

Cancer pain is caused by the tumor itself, bone invasion, compression of the spinal cord or nerve structures, and pressure from hollow organs. Morphine is currently used to treat both acute and chronic pain. However, its use is hampered by apparent analgesic tolerance and other adverse effects, and the unknown mechanism of the resistance action remains a major challenge.

Generally, neuropathic pain caused by malignant tumor compression or infiltration of peripheral nerves predominates in patients with cancer among three conditions: somatic, visceral, and NCP 32. In this study, we found that a cancer pain model using an intramuscular injection of S-180 cells into the sciatic nerve was a consistent short-term animal model. This model can mimic certain clinical manifestations of cancer pain—such as nerve compression, sensory impairment, and spontaneous pain—and can thus be used to study cancer pain treatment. In our study, cancer pain was characterized by lifting, shaking, and licking of the right hind limb, accompanied by foot dragging, jumping gait, and disappearance of the claw extension reflex. While walking on the cage ceiling, the right foot of the mouse held an obstacle that was easily emptied and leaked through the mesh. As the tumor grew, the mice gradually showed sensory deficits and frequently showed spontaneous pain symptoms, such as lifting of the right hind limb, trembling, and no weight bearing. By day 22, the sciatic nerve was almost completely surrounded by the S-180 tumor mass, and mice in the model group showed protective lifting of the affected side to avoid touching the ground. In the pain behavior test, some mice with transplanted tumors for over 22 days did not respond to pressure, which could be due to nerve paralysis. Similar phenomena can also occur in patients with advanced cancer 33,34, further reinforcing the success of this experimental model.

APLNR is present in the human cardiac and dentate myocytes and vascular endothelial cells. The apelin (endogenous ligand of APLNR)/APLNR system is involved in various physiological and pathological processes, including cardiovascular disease, angiogenesis, energy metabolism, and humoral homeostasis 35. The apelin/APLNR system exerts dual effects on acute inflammatory, and neuropathic pain. The APLNR antagonist ML221 reduces pain hypersensitivity induced by chronic systolic injury and inhibits ERK phosphorylation in the spinal dorsal horn 36. Apelin (intracerebroventricular injection, 0.4 μmol/rat) reduced the pain threshold in the rat tail flapping experiment 36. The contradictory results regarding the role of apelin/APLNR in pain modulation are difficult to explain. It may be related to the type of pain, dose, type of animal, route of administration, and time of injection in the animal models. The main molecular mechanisms underlying apelin/APLNR-induced pain are related to opioid receptors, γ-aminobutyric acid receptors, and the ERK pathway 37. Here we found that long-term injection of morphine in mice leads to the morphine metabolite M3G accumulation, which activates ERK1/2 via APLNR and ultimately activates the release of microglia and inflammatory factors TNF-α, IL-1β, and IL-17, exacerbating NCP. These findings add to our understanding of the role of APLNR in pain and highlight the important mechanisms of morphine tolerance. We also found that M3G binds to the MOR and activates ERK1/2, in addition to activating ERK1/2 through APLNR. Morphine has two metabolites: M3G and morphine-6-glucuronide (M6G). M6G binds to the opioid receptors and exerts analgesic effects. M3G has low affinity for opioid receptors and may be involved in the development of morphine tolerance 38. Experiments showed that M3G can activate ERK1/2 and microglial proliferation to some extent. Compared to this, the effect of M3G binding and acting with APLNR is more obvious; therefore, it can be hypothesized that in morphine tolerance, M3G may act more through APLNR and only slightly or through MOR to some extent.

S-Palmitoylation (palmitoylation of cysteine) is a reversible post-translational modification mediated by the DHHC family of palmitoyl transferases and is reversed by several acyl-protein thioesterases 19, 39. Although S palmitoylation occurs in thousands of human proteins, little is known about the how it regulates specific biological functions. Recent studies have suggested that members of the DHHC family are involved in inflammatory responses in organ failure. Functional impairment of ZDHHC21 resulted in significant resistance to injury, characterized by reduced plasma leakage, reduced leukocyte adhesion, improved lung pathology, and—ultimately—improved survival 40. ZDHHC7 palmitoylates STAT3 and promotes the membrane recruitment, phosphorylation, and differentiation of TH17 cells 41. We found that ZDHHC5, 9, 17, 19, and 23 were upregulated in the NCP model, indicating functional redundancy in the regulation of protein palmitoylation during cancer-induced pain or demonstrating the complexity of protein palmitoylation regulation in response to different stimuli. Consistent with this, the types and abundance of palmitoylated proteins increased after NCP onset. Notably, ZDHHC9 specifically increased the palmitoylation of APLNR to prevent its degradation by the lysosomal pathway. M3G, the main metabolite of morphine, accumulated in the cancer pain model and activated ERK1/2 signal transduction through APLNR. ERK1/2 further increases the expression of ZDHHC9 and the stability of APLNR. Thus, there is a positive feedback loop for ZDHHC9, ERK1/2, and APLNR in the NCP model that exacerbates pain-induced inflammatory responses and drug resistance in cancer. Additionally, we found that in the NCP model, palmitoylation of the astrocyte marker protein GFAP is upregulated, promoting the proliferation of glial cells and the inflammatory response and participating in the signaling and maintenance of cancer pain together with microglia (data not shown). In fact, altered levels of protein palmitoyl modification or palmitoyltransferase expression were detected to varying degrees in both CFA- and SNI-induced models of pathological pain, suggesting that abnormal or altered palmitoyl modification may be a common phenomenon in pathological pain and so could be a potential target for clinical treatment (data not shown).

The mature lipid 2-BP is a non-specific inhibitor 42. It blocks the palmitoyltransferase activity of all the DHHC proteins previously evaluated, increasing the risk of unknown side effects 43. Although competitive inhibition effectively targets specific enzymes, it is not widely used to inhibit DHHC acetyltransferases. Having identified the palmitoylation motif of APLNR, we designed a short substrate sequence to competitively inhibit the palmitoylation of endogenous APLNR. This competitive peptide, which targets the APLNR palmitoylation site in combination with morphine, can inhibit the development of NCP, including pain incidence, microglial activation, and inflammatory factor release and alleviate morphine tolerance.

## Conclusions

In summary, during the occurrence and development of NCP, the expression of palmityltransferase ZDHHC9 was increased, and palmitylated APLNR promoted its stability. The use of morphine can lead to the accumulation of the metabolite M3G *in vivo*, and through APLNR, the ERK1/2 signaling pathway, leading to the activation of microglia, the release of inflammatory factors, and finally, the formation of morphine tolerance. Thus, targeted palmitoyl modification of APLNR seems to be an effective method for treating cancer pain.

## Supplementary Material

Supplementary methods and figures.

## Abbreviations

BP: 2-bromopalmitate
ABE: acyl-biotin exchange assay
APLNR: apelin receptor
CB1: cannabinoid receptor 1
ELISA: enzyme linked immunosorbent assay
GABAB: gamma-aminobutyric acid (GABA) B receptor 1
HAM: Hydroxylamine
Iba1: induction of brown adipocytes 1
IL: interleukin
LC-MS/MS: liquid chromatography-triple quadrupole mass spectrometry
M3G: morphine-3-glucuronide
MOR: mu opioid receptors
MTNR1A: melatonin receptor 1A
NCP: neuropathic cancer pain
NK1R: tachykinin receptor 1
PWT: paw withdrawal threshold
qRT-PCR: quantitative real-time PCR
TNF-α: tumor necrosis factor-alpha
RT-PCR: real-time polymerase chain reaction
ZDHHC: zinc finger DHHC domain-containing protein
