# Supplementary material for: Blocking Palmitoylation of Apelin Receptor Alleviates Morphine Tolerance in Neuropathic Cancer Pain
Source: Int J Biol Sci. 2024 Jan 1;20(1):47–60. doi: 10.7150/ijbs.86888 (PMC10750292; doi:10.7150/ijbs.86888)
Supplement: Supplementary file 1 — Supplementary methods and figures. [file ijbsv20p0047s1.pdf]

SUPPLEMENTARY INFORMATION

Title

Blocking Palmitoylation of Apelin Receptor Alleviates Morphine Tolerance in Neuropathic Cancer Pain

Author information

Xiaoqing Fan, Ph.D.,[1,2,3,4] Meiting Gong, M.D.,[1,3,5] Siyu Zhang, M.D.,[1,2] Wanxiang Niu, Ph.D.,[1,2] Suling Sun, Ph.D.,[1,2] Huihan Yu, M.D.,[1,3,5] Xueran Chen, Ph.D.,[1,2,3,*] Zhiyou Fang, Ph.D.,[1,2,3,*]

[1]Anhui Province Key Laboratory of Medical Physics and Technology; Institute of Health and Medical Technology, Hefei Institutes of Physical Science, Chinese Academy of Sciences, No. 350, Shushan Hu Road, Hefei, Anhui, 230031, China

[2]Science Island Branch, Graduate School of University of Science and Technology of China, No. 96, Jin Zhai Road, Hefei, Anhui, 230026, China

[3]Department of Laboratory Medicine, Hefei Cancer Hospital, Chinese Academy of Sciences, No. 350, Shushan Hu Road, Hefei, Anhui, 230031, China

[4]Department of Anesthesiology, The First Affiliated Hospital of USTC, Division of Life Sciences and Medicine, University of Science and Technology of China (USTC), No. 17, Lu Jiang Road, Hefei, Anhui, 230001, China

[5]Department of Pathophysiology, School of Basic Medicine, Anhui Medical University, No. 81, Meishan Road, Hefei, Anhui, 230032, China

***Corresponding author**: Dr. Xueran Chen (xueranchen@cmpt.ac.cn), and Prof. Zhiyou Fang (z.fang@cmpt.ac.cn).

Supplementary information for Methods

Antibodies and reagents

Antibodies against Iba1 (17198), ERK1/2 (9102), *p*-ERK1/2 (4370), HA (3724), and β-actin (3700) were purchased from Cell Signaling Technology (Colorado, USA). Antibody against APLNR (sc-517300) was purchased from Santa Cruz Biotechnology (California, USA). Antibodies against ZDHHC9 (ab74504) were purchased from Abcam (Cambridge, UK). AlexaFluor488 goat anti-rabbit immunoglobin (Ig)G (A11008) and AlexaFluor568 goat anti-rabbit IgG (A11011) were purchased from Invitrogen (California, USA). Rhodamine phalloidin (PHDR1) was purchased from Cytoskeleton (Colorado, USA).

2-bromopalmitate (2-BP) (21604) was purchased from Sigma-Aldrich (Missouri, USA). N-Ethylmaleimide (23030), hydroxylamine HCl (HAM) (26103), and EZ-Link™ HPDP-biotin (A35390) were purchased from Thermo Fisher Scientific (Massachusetts, USA). Lysosomal inhibitors, leupeptin (HY-18234A) and bafilomycin A1 (HY-100558), and proteasome inhibitor, MG132 (HY-13259), were purchased from MedChemExpress (New Jersey, USA).

Cell culture and plasmids

Mouse sarcoma S-180 cells and microglial BV2 cells were obtained from the Cell Bank of Type Culture Collection of the Chinese Academy of Sciences, Shanghai Institute of Cell Biology, Chinese Academy of Sciences (Shanghai, China), where they were characterized by deoxyribonucleic acid (DNA) fingerprinting and isozyme detection. Cell lines were cultured in Dulbecco's modified eagle medium (Gibco BRL, New York, USA) supplemented with 10% fetal bovine serum and 1% (100×) penicillin/streptomycin (Gibco) in the presence of 5% CO_2 at 37°C.

APLNR (C325, 326A) mutant plasmids were generated by site-directed mutagenesis using the Fast Mutagenesis System (FM111-01) from TransGen Biotech (Beijing, China). All plasmids were verified by DNA sequencing.

The Knockout kit (CRISPR) for targeting ZDHHC9 (KN400083), APLNR (KN407576), CB1 (KN410397), GABAB (KN408503), NK1R (KN410395), MTNR1A (KN410385), MOR (KN410383), and non-specific control sequences was purchased from Origene (Maryland, USA). To evaluate the gene editing activity of g-ribonucleic acid (RNA), the genomic DNA of gRNA-transfected cells was extracted, and these genes were amplified. Cells were cultured in a medium containing 0.5 μg/mL puromycin, which was selected, passaged, and confirmed via DNA sequencing.

Western blotting and immunoprecipitation

After mice were euthanized by intraperitoneal injection of over dose pentobarbital (150 mg/kg), L4–L6 spinal dorsal horns were collected and lysed. The lysates were centrifuged at 12,000 × g at 4°C. The supernatant was collected, and the total protein concentration was quantified using a bicinchoninic acid protein assay kit (Thermo Fisher Scientific, Massachusetts, USA). Equal amounts of protein were separated on a 10% polyacrylamide gel (Bio-Rad, California, United States). Proteins were then transferred onto polyvinylidene difluoride membranes. Membranes were blocked in 5% non-fat milk for 2 h at room temperature and incubated with the primary antibodies anti-Iba1 (1:2000), anti-ERK1/2 (1:2000), anti-*p*-ERK1/2 (1:1000), anti-APLNR (1:500), and anti-β-actin (1:2500) at 4°C overnight. After being washed with phosphate buffered saline (PBST) (0.5% Tween-20), membranes were incubated with peroxidase-conjugated secondary antibodies for 1 h at room temperature. An imaging system (Bio-Rad, California, USA) was used to examine chemiluminescence. Protein expression was normalized to glyceraldehyde 3-phosphate dehydrogenase expression.

For immunoprecipitation, western blotting and immunoprecipitation lysates (20 mM Tris [pH 7.5], 150 mM NaCl, 1% Triton X-100, sodium pyrophosphate, β-glycerophosphate, EDTA, Na_3VO_4, and leupeptin) (Beyotime, Shanghai, China) with a cocktail (Roche, Basel, Switzerland) were used to prepare protein extracts. To every 500 μg of lysate, 1 μg of the corresponding antibody was added and combined overnight at 4°C. The mixture was incubated with 30 μL protein A/G beads at room

temperature for 2 h. After the bonded beads were washed with PBST five times, 30 μL of sodium dodecyl sulfate (SDS) sample buffer was added for western blot analysis.

Real-time quantitative polymerase chain reaction

Total RNA was extracted from the L4–L6 spinal dorsal horn using TRIzol (Invitrogen, California, USA). cDNA was synthesized from total RNA using the First-strand cDNA Synthesis SuperMix kit (TransGen Biotech, Beijing, China), according to the manufacturer's instructions. The primer sequences are as follows: *ZDHHC5*, forward, 5'-acacctcggcttggctacta-3' and reverse, 5'-gttggctccttcaagctgtc-3'; *ZDHHC9*, forward, 5'-ctttcctcgtggctctcaac-3' and reverse, 5'-tcctccagtggcaaaatacc-3'; *ZDHHC16*, forward, 5'-aaggagagacgtcggctaca-3' and reverse, 5'-cacagaggctgagtgagcag-3'; *ZDHHC17*, forward, 5'-ctccacccagaggaaatcaa-3' and reverse, 5'-gttattgatggcagcccaat-3'; *ZDHHC18*, forward, 5'-tgtggggagacggaactatc-3' and reverse, 5'-acgtgtgaaaccctgagagg-3'; *ZDHHC19*, forward, 5'-tctttgctgccttcaatgtg-3' and reverse, 5'-agcggagccttgatgtaaga-3'; and *ZDHHC23*, forward, 5'-tgcctgtcttccttcatgtg-3' and reverse, 5'-agcccagagagaacagtcca-3'. To evaluate gene expression, $2^{-\triangle\triangle Ct}$ values were calculated. mRNA expression levels were normalized to those of β-actin.

Immunofluorescence assay

Mice were anesthetized using an intraperitoneal injection of pentobarbital (150 mg/kg) and transcardially perfused with saline solution, followed by 4% paraformaldehyde. The L4–L6 spinal dorsal horn was then removed, fixed in paraformaldehyde, and dehydrated in 10%, 20%, and 30% sucrose (Sinopharm Chemical Reagent Co. Ltd, Beijing, China.) in succession until they sank and cut into 12-μm sections using a freezing microtome (Leica, Wetzlar, Germany). Sections were blocked with 4% normal goat serum, followed by incubation with primary antibodies: anti-Iba1 antibody (1:500) at 4°C overnight. After being washed with PBST, secondary antibodies labeled AlexaFluor 488 or 555 (Molecular Probes, New York, USA), were incubated at room temperature for 40 min. Slides were observed under a fluorescence

microscope (Leica, Wetzlar, Germany). Negative controls were prepared by omitting the primary antibodies.

Enzyme-linked immunosorbent assay (ELISA)

ELISA was performed in accordance with the instructions of the manufacturer. The wild-type, APLNR-knockout, or ZDHHC9-knockdown microglia BV2 cells was treated with morphine-3-glucuronide (M3G) (5 μM) for 24 hours, and culture supernatant were collected and homogenized and measured the expression of TNFα (MTA00B, R&D system), IL-1β (MLB00C, R&D system), and IL-17 (M1700, R&D system) by ELISA kits.

Cell viability assay

The cells were seeded in 96-well plates at 4,000 cells/well and incubated overnight. A Cell Counting Kit-8 assay (Beyotime, Shanghai, China) was used to detect cell viability. Absorbance was measured at a wavelength of 450 nm. All experiments were performed in triplicate.

Supplementary information of Figures

Supplementary Figure 1

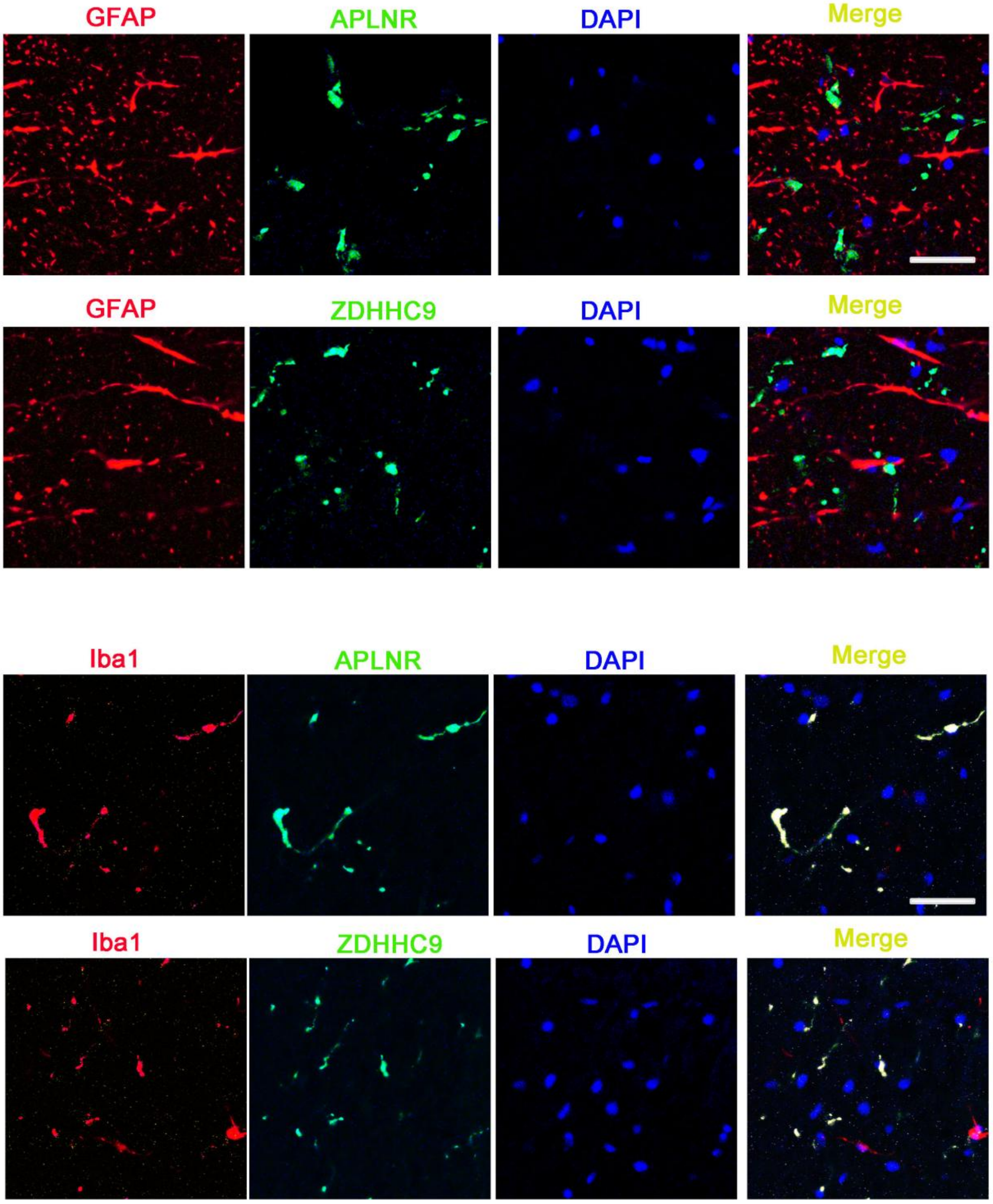

Supplemental Figure 1. Co-localization of Iba1 and APLNR (or ZDHHC9) in neuropathic cancer pain (NCP) mice.

Immunofluorescence analysis of Iba1 (microglial marker) and APLNR (or ZDHHC9) expression, or GFAP (astrocyte Marker) and APLNR (or ZDHHC9) expression in the L4–6 spinal cord horn of NCP groups.

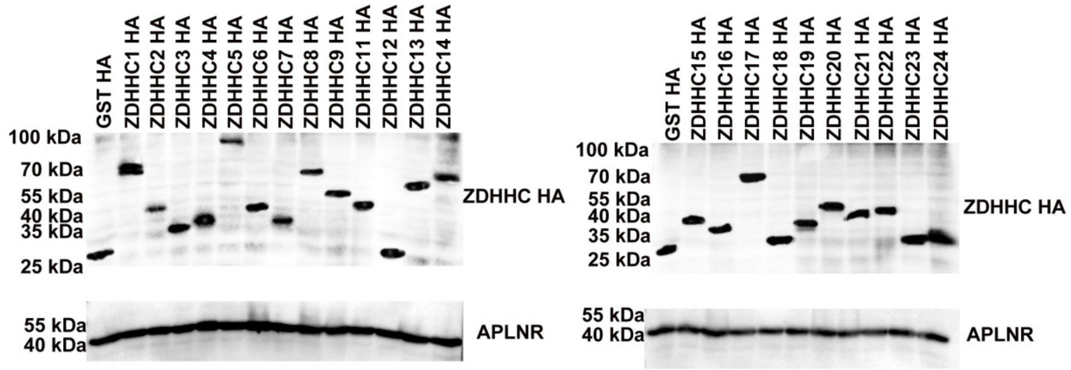

Supplemental Figure 2. APLNR interacted with DHHC family.

APLNR was immunoprecipitated from BV2 cells transfected with 23 HA-tagged ZDHHCs using an anti-HA antibody, and detected by immunoblotting with the APLNR antibody.
